# Thermoset Polyester Resin Microplastics: Effects on Enzymatic Biomarkers and Toxicological Endpoint Responses of *Eisenia fetida* Earthworms

**DOI:** 10.3390/toxics13070602

**Published:** 2025-07-17

**Authors:** David Amaya-Vías, Gemma Albendín, Vanessa Aranda-Quirós, Rocío Rodríguez-Barroso, Dolores Coello, Juana María Arellano

**Affiliations:** 1 Área de Toxicología, Faculty of Marine and Environmental Sciences, Instituto Universitario de Investigación Marina (INMAR), Campus de Excelencia Internacional del Mar (CEIMAR), University of Cadiz, Av. República Saharaui, Puerto Real, 11510 Cadiz, Spain; david.amaya@uca.es (D.A.-V.); vanessa.aranda@uca.es (V.A.-Q.); juana.arellano@uca.es (J.M.A.); 2 Departamento de Tecnologías del Medio Ambiente, Faculty of Marine and Environmental Sciences, Instituto Universitario de Investigación Marina (INMAR), Campus de Excelencia Internacional del Mar (CEIMAR), University of Cadiz, Av. República Saharaui, Puerto Real, 11510 Cadiz, Spain; rocio.rodriguez@uca.es (R.R.-B.); dolores.coello@uca.es (D.C.)

**Keywords:** thermoset microplastics, polyester resins, biomarkers, *E. fetida*

## Abstract

Thermosets are plastic composite materials widely used in many industrial sectors of modern society with an increasing presence in the environment. The adverse effects of this material on environmental compartments and biota of thermosets are still unknown. In this work, we studied the potential effects of two thermoset polyester resin-derived microplastics (R930A-SP and R930A-DVE1) on the survival, behavior, morphological changes and subcellular damage of earthworms *Eisenia fetida*. The proposed experimental conditions simulated environmentally relevant concentrations, taking as a reference other related microplastics present in the environment. Thus, *E. fetida* specimens were exposed to five concentrations (100, 500, 1000, 1500 and 2000 mg resin per kg soil) of these two resins for 14 days. At concentrations and exposure times studied, no significant effects on growth, measured as weight loss, or on the enzyme biomarkers (cholinesterase, carboxylesterase and glutathione S-transferase) were observed. Similarly, no behavioral changes were detected in earthworms, and the survival rate was 100%. Likewise, no differences were observed between the different formulations of the polyester resins studied. However, this study could serve as a starting point for further studies with higher concentrations and/or exposure times, as well as in combination with other pollutants.

## 1. Introduction

Plastic pollution is currently one of the main issues that potentially affect the global health of the environment, ecosystems and human well-being [1,2,3,4]. Plastic materials are composed of a large mixture of substances and additives and cannot be classified according to a single point of view. Thus, some plastic classifications exist according to their physical properties, size, main polymers and chains, manufacturing characteristics or product purpose [5]. Most plastic materials are not biodegradable and recyclable, and end-of-life treatment mainly involves incineration, landfill or direct release into the environment, which is one of their main drawbacks [5,6]. Consequently, plastic pollution is widespread in all environmental compartments, even reaching remote areas [5,7,8]. Plastics are considered persistent substances and emerging contaminants, degrading slowly into microplastics (1–5000 µm) and nanoplastics (<1 µm) and releasing various residual chemicals into the environment as additives, monomers, oligomers and polymers [5,8,9,10,11]. Therefore, from the point of view of studying potential hazardous effects, toxicity assessment is challenging due to the unknown and changing mixture of chemical compounds from plastics released into the environment [6,8,10,11,12,13]. One of these plastic materials are polymer composite materials or just composites. These materials, which are composed of polymers reinforced with fibers or other materials, have been used for a long time [14]. However, due to the development of the crude oil industry and the chemical industry of synthetic polymers, their use has increased exponentially since the middle of the 20th century [15,16]. These materials are essential for industry and engineering due to their specific characteristics: design, manufacturing, mechanical properties, high performance and low weight. Nowadays, composites can be found in numerous applications (chemical, electronic, electrical, adhesives, paints and coatings, among others) in sectors such as construction, energy, automotive, aerospace or marine, and are key to maintaining the standards of modern society [15,16,17,18]. In fact, the per capita use of composites is currently considered an indicator of technological development [15]. In recent decades, a wide range of new composites have been developed according to the specific requirements needed for each application. In this respect, polyester resins are particularly noteworthy. These resins are part of thermoset composites, together with epoxy or vinyl ester resins. Thermosets are liquid or solid substances with a low melting point that, once cured (by catalysis reactions, heat or a combination of both), cannot return to their original form and cannot melt or remolded, unlike thermoplastics [15]. In terms of composition, polyester resins contain long linear polymeric chains (unsaturated polyesters) dissolved in a vinyl monomer, usually styrene [14,15,16]. Styrene, in proportions up to 50% (typically 35–45%), provides high corrosion resistance and reduces the viscosity of the resin, making it easier to handle [15,16,19]. In addition, styrene plays an essential role, as it allows the cross-linking of polyester chains. This viscous liquid is then cured, usually with methyl ethyl ketone peroxide (MEKP) and a catalyst such as cobalt naphthenate [14]. These polyester resins are much less expensive than epoxy resins and are easy to produce and handle. They have low weight and balanced mechanical, chemical and electrical properties, which make them attractive for marine, electrical, chemical and automotive applications [14,15]. From a toxicological point of view and despite all these advantages, it should be noted that some of the components of polyester resins could be harmful to the environment and biota, such as styrene [19,20,21,22]. Styrene is identified as a volatile organic compound (VOC) and a hazardous air pollutant (HAP), according to the Environmental Protection Agency of the United States (US EPA) [23]. In addition, as of 2019, styrene is considered by the International Agency for Research on Cancer (IARC) as a probable human carcinogen (Group 2A) [24]. Likewise, because of the inherently complex nature of polyester resins and thermosets in general, these materials generally are not suitable for recycling at the end-of-life stage, and reuse is very limited [6,16,17,18]. Currently, waste from these materials is incinerated or landfilled [6,17,25]. Approximately 306,000 tons of composite waste are generated and accumulated each year worldwide [26]. Furthermore, it is unknown exactly how the ingredients of composites may migrate or be released after incineration or storage into the environmental compartments [6,8]. Further toxicological studies are also required to determine the actual interactions, hazards and toxicity of these components in the environment, biota and humans, as some of them are known to be mutagenic and carcinogenic in animals and humans, such as the aforementioned styrene [6,17,19,20,21]. For this reason, in recent years, the research has focused on two main lines [17]: on the one hand, the development of new, more sustainable technologies for the treatment, recycling and valorization of these materials [17,25,26]; and on the other hand, the efficient development of new bio-based, less toxic materials and their inclusion in traditional formulations [17,18,19,20,21,27,28,29,30]. In this sense, it is worth highlighting some international projects financed by the European Union that work in this direction, such as the BARBARA project [31], the LIFE RECYSITE project [32], the VIBES project [18,33] or the BIZENTE project [25,26,34], among others. At this point, toxicity assessments are needed to provide more information on the effects of these substances in the environment, especially in one of the environmental compartments initially most affected, the soil [8,35,36]. One of the most representative and abundant soil organisms are earthworms. Earthworms play an essential role in terrestrial ecosystems and are excellent soil quality bioindicators [37,38,39]. In particular, earthworms influence the physicochemical characteristics of soils by burrowing, mixing the different soil layers, transferring and transforming organic matter and other substances [8,39]. Thus, these organisms improve the structural integrity, stability, aeration and irrigation of soils and are considered soil engineers [37]. *Eisenia fetida* is considered a model species in toxicology, as outlined in the OECD guidelines, allowing the identification of the potential toxicity of new pollutants reaching terrestrial systems [40,41,42]. Furthermore, the analysis of different endpoints such as mortality, growth or reproduction in earthworms, in combination with biomarkers, provide very useful information to understand the effects of pollutants in soil [38,41,42]. Some recent studies have been carried out with earthworms (*Eisenia fetida*, *Eisenia andrei* and *Lumbricus terrestris*) and micro- and nanoplastics (polyethylene, polystyrene and polypropylene) under different experimental conditions [35,36,39,43,44,45,46,47]. Different neurotoxicity and oxidative stress effects have been found in some of these toxicity studies [36,39,44], especially for long-term exposure setup and high microplastic concentrations [8]. Microplastics can obstruct the digestive tract of earthworms, cause some histological damage to tissues and reduce the growth rate of these organisms [8,35,45,46]. However, no works have yet been found on the effects of thermosets such as polyester resins on earthworms or similar organisms. This work aims to provide the first overview of the effects of two polyester resins on the earthworm *Eisenia fetida* at realistic experimental conditions and simulating environmentally relevant concentrations. The two R930A polyester resins are mainly used as flame protection coatings. One resin contains a commercial composition, with polyester polymers dissolved in styrene. Meanwhile, styrene has been replaced in the other resin by an alternative, more sustainable, synthesized compound (DVE-1). Responses of *Eisenia fetida* were determined by survival rate, behavioral changes and weight changes as primary endpoints at an individual level. In addition, subcellular level changes were assessed by enzymatic biomarkers of neurotoxicity, such as cholinesterase (ChE), and of detoxification and oxidative stress, such as carboxylesterase (CbE) and glutathione S-transferase (GST).

## 2. Materials and Methods

### 2.1. Chemicals and Reagents

The chemicals used were as follows: di-sodium hydrogen phosphate anhydrous (Na_2_HPO_4_, CAS 7558-79-4, 99% ACS, Merck, Darmstadt, Germany), sodium dihydrogen phosphate monohydrate (NaH_2_PO_4_×H_2_O, CAS 10049-21-5, ACS, Merck, Darmstadt, Germany), Tris (hydroxymethyl) amino-methane (C_4_H_11_NO_3_, CAS 77-86-1, ≥99.8% ACS, Sigma-Aldrich/Merck, Darmstadt, Germany), hydrochloric acid (HCl, CAS 7647-01-0, 37% ACS, Scharlau, Barcelona, Spain), 5,5′-Dithiobis (2-nitrobenzoic acid) (DTNB—C_14_H_8_N_2_O_8_S_2_, CAS 69-78-3, ≥98% TLC, Sigma-Aldrich/Merck, Darmstadt, Germany), acetylthiocholine iodide (C_7_H_16_INOS, CAS 1866-15-5, ≥98% TLC, Sigma-Aldrich/Merck, Darmstadt, Germany), 4-Nitrophenyl valerate (C_11_H_13_NO_4_, CAS 1956-07-6, ≥98% TLC, Sigma-Aldrich/Merck, Darmstadt, Germany), acetone (C_3_H_6_O, CAS 67-64-1, ≥99.5% ACS, Honeywell|Riedel-de Haën™, Seelze, Germany), L-Glutathione, reduced (GSH—C_10_H_17_N_3_O_6_S, CAS 70-18-8, 99%, Sigma-Aldrich/Merck, Darmstadt, Germany), 1-Chloro-2,4-dinitrobenzene (CDNB—C_6_H_3_C_l_N_2_O_4_, CAS 97-00-7, ≥99%, Sigma-Aldrich/Merck, Darmstadt, Germany), ethanol absolute (C_2_H_6_O, CAS 64-17-5, 99.8% ACS, Scharlau, Barcelona, Spain), bovine serum albumin (BSA, Bio-Rad Protein Assay Standard II, Hercules, CA, USA), dye reagent (Bio-Rad Protein Assay Dye Reagent Concentrate, Hercules, CA, USA), and ultrapure water (Synergy^®^, Merck Millipore, Darmstadt, Germany).

### 2.2. Polyester Resins

The R930A resins under study were synthesized and provided by Specific Polymers, France. From unpublished data, one of the resins, named R930A-SP, contains a formulation like a commercial R930A resin using styrene. In the other resin, named R930A-DVE1, non-degradable styrene is replaced by a synthesized divinyl ester (DVE-1) as an alternative compound.

Both resins, previously ground and sieved, had a particle size between 1000 µm and 355 µm. Thus, resins were within the particle size range of the soil used (<2000 µm). According to the OECD guideline No. 207 [40] with changes as described below and studies on earthworm exposure to microplastics at environmentally relevant concentrations [33,36,42,43], five concentrations were established for both resins, as follows: 100, 500, 1000, 1500 and 2000 mg resin per kg soil (0.02–0.2% *w*/*w*). The resin amount added to each test jar was weighed with an analytical balance (Sartorius ED124S, d = 0.1 mg). All tests were carried out in triplicate.

### 2.3. Substrate Matrix

The substrate selected for the tests was a commercial substrate classified as suitable for organic farming. According to the manufacturer (Grandiol—Lidl Supermercados S.A.U, Barcelona, Spain), the substrate composition was 38% vegetable compost, 30% coconut fiber, 15% composted pine bark, 15% Sphagnum peat and 2% perlite. Regarding physical–chemical characteristics, the manufacturer reported an organic matter content of 65% of dry matter, electrical conductivity of 25 mS/m, pH 7.6, particle size <0.20 cm and dry bulk density of 348 kg/m^3^.

To ensure substrate homogeneity and characteristics such as moisture, all tests were carried out with the same container, at the same time and under the same environmental conditions. Therefore, a total of 13.2 kg of substrate was used in the experiments. Each of the test jars contained 400 g of substrate, weighed with a precision balance (Cobos C-1800-CS, d = 0.01 g) and subsequently mixed with the corresponding quantities of polyester resins 24 h before starting the experiments.

### 2.4. Test Organism

The organism used in this study was the epigean earthworm, *Eisenia fetida*, supplied by a local earthworm farm. All earthworms were previously acclimatized to environmental conditions and domesticated for 15 days in the test soil with adequate feeding and humidity. All exposure tests were conducted on randomly selected adults, with well-developed clitellum and similar size with a mean (±SD) weight of 0.278 ± 0.06 g.

Just 24 h before the start of the experiments, 165 earthworms were selected and placed individually in amber-colored glass jars to protect them from light. These jars contained only a piece of cotton wool moistened with 5 mL of tap water to reduce the risk of osmotic shocks in the organisms. The volume of water added and its characteristics (tap water instead of ultrapure water) were previously optimized to prevent excessive or deficient humidity and stress on the organisms. This process aims to empty the earthworms’ gut contents, favoring a stronger appetence for the test soil, as reported in different studies [39,44,45,46,47,48,49].

After the depuration period, earthworms were rinsed with ultrapure water to remove any remaining soil and feces. Subsequently, earthworms were dried carefully using absorbent paper, removing only the water surplus. Before placing the organisms into the testing jars, the earthworms were weighed using a precision balance (Sartorius TE153S d = 0.001 g), recording the individual fresh weights at the start of the assay.

### 2.5. In Vivo Acute Toxicity Test

The entire exposure test was carried out in 1L clear glass jars. Jars with each resin concentration were homogenized and shaken vigorously to ensure uniform distribution of the particles in test soil. Additionally, a control was included containing just the substrate. All tests were carried out in triplicate.

Then, to maintain adequate substrate moisture for a few days, 250 mL of ultrapure water was added to each jar. Immediately afterwards, 5 earthworms, previously purged, washed and weighed, were also added to each exposure condition and replicate. All earthworms were quickly hidden in the soil within a few minutes without signs of lethargy.

According to the OECD guideline No. 207 for acute toxicity test in earthworms, the experiments were performed for 14 days [40]. All jars were kept in a laboratory with environmental conditions of photoperiod (light–dark cycles of approximately 12:12 h, no direct sunlight) and temperature. The average temperature was 25 °C, with a maximum of 27 °C and a minimum of 22 °C for the 14 days. The jars were non-hermetically closed to allow enough ventilation, and the earthworms were not fed to increase substrate consumption. Substrate temperature and humidity were checked daily. In addition, any morphological and behavioral changes (lethargy, earthworms on the surface or attached to the cover, physiological damage, coiling or expulsion of coelomic fluid and mucus, among others) of the earthworms were monitored daily and mainly on days 7 and 14.

On day 7, a mortality count was carried out. The contents of each of the jars were carefully checked in laboratory trays. The surviving earthworms and the substrate with the resin mixture were returned to the jars, without any sample loss. In this respect, substrate moisture was corrected by adding 100 mL of ultrapure water to each container. Finally, on day 14, a new mortality count was performed again. Surviving earthworms were identified and purged in the same way as at the start of the assay. The weight of the surviving earthworms after 24 h of purging corresponds to the individual fresh weight at the end of the experiment.

### 2.6. Sample Preparation

The purged and weighed surviving earthworms were individually placed into labeled microcentrifuge tubes. The tubes were immediately placed on ice. Subsequently, as soon as all microtubes were prepared, the earthworms were sacrificed in a −20 °C freezer and stored there until homogenization as proposed in different studies [48,50].

For the homogenization procedure, the whole earthworm was placed in ice-cold 0.1 M phosphate buffer pH 7.4 at a ratio of 20 mg tissue per mL buffer. The homogenates were centrifuged at 10,000 min^−1^ (9000× *g*) at 4 °C for 30 min using a Heraeus sepatech Megafuge 1.0 R centrifuge (Thermo Fisher Scientific, Madrid, Spain). Supernatants (hereafter referred to as samples) were collected in fresh tubes and stored at −20 °C until further protein and biomarker analysis.

### 2.7. Enzymatic Biomarker Measurements

Based on previous unpublished optimization studies, earthworm homogenate in 0.1 M phosphate buffer pH 7.4 was used for all biomarker and protein measurements. In this regard, all measurements were performed in triplicate, including a reagent blank, in 96-well microplates using a Tecan Infinite M-Nano microplate reader. All enzyme activities were expressed in terms of specific activity (nmol min^−1^ mg protein^−1^).

#### 2.7.1. Protein Content Determination

Protein contents were estimated by the Bio-Rad protein assay, based on the method of Bradford (1976) [51] and adapted to microplates. The calibration curve was performed using bovine serum albumin (BSA) as a standard, and protein concentration was calculated accordingly. Protein concentrations of the samples were used to calculate the specific enzyme activities.

#### 2.7.2. Cholinesterase (ChE) Activity

The procedure used to determine ChE activity is based on the method reported by Ellman et al. (1961) [52], adapted to microplates as described by Albendín et al. (2021) [53] with modifications.

Firstly, just before the assay, a reaction mixture was prepared containing 30 mL of 0.1 M phosphate buffer pH 7.4, 1 mL of 0.01 M 5,5′-Dithiobis (2-nitrobenzoic acid) (DTNB) in phosphate buffer and 200 µL of 0.2 M acetylthiocholine iodide substrate in ultrapure water. Then, 250 µL of this reaction mixture was added to 50 µL of sample in each well. The ChEs activity was measured immediately at 415 nm, every 30 s for 3.5 min at room temperature.

#### 2.7.3. Carboxylesterase (CbE) Activity

The procedure used to determine CbE activity is based on the method reported by Carr and Chambers (1991) [54], adapted to microplates as described by Soto-Mancera et al. (2020) [55] with modifications.

A stock solution of 4-Nitrophenyl valerate 100 mM was prepared in acetone as substrate. From this, a 2 mM working solution was prepared in ultrapure water. To each well, 20 µL of sample, 80 µL of 0.05 M Tris-Cl buffer pH 7.5 and 100 µL of 2 mM 4-Nitrophenyl valerate substrate (final well concentration of 1 mM) were added. The CbE activity was measured immediately at 405 nm, every 30 s for 3 min at room temperature.

#### 2.7.4. Glutathione S-Transferase (GST) Activity

The procedure used to determine GST activity is based on the method reported by Habig et al. (1974) [56], adapted to microplates as described by Frasco and Guilhermino (2002) [57] with modifications.

A reaction mixture was prepared just before the assay containing 4.95 mL of 0.1 M phosphate buffer pH 6.5, 0.15 mL of 60 mM 1-Chloro-2,4-dinitrobenzene (CDNB) in ethanol and 0.9 mL of 10 mM L-Glutathione (GSH) substrate in phosphate buffer. Then, 200 µL of this reaction mixture was added to 100 µL of sample in each well. The GST activity was measured immediately at 340 nm, every 20 s, for 5 min at room temperature.

### 2.8. Data Analysis

Data collection and processing were carried out using Microsoft^®^ Excel^®^ 2019. Unless otherwise stated, data are expressed as the mean ± standard error of the mean (SEM). For the statistical analysis, IBM^®^ SPSS^®^ Statistics v.25.0 was used. Normality and homoscedasticity of data were assessed using the Shapiro–Wilk and Levene tests. Significant differences between treatments and control groups for each of the biomarkers and endpoints were studied by analysis of variance (one-way ANOVA). When the assumption of normality was not satisfied, the Kruskal–Wallis test was used to determine whether there was any significant difference. Significant differences between groups were accepted for *p*-values less than 0.05.

## 3. Results and Discussion

### 3.1. Individual Endpoints: Survival Rate, Morphological Changes and Behavioral Changes

Among the most direct endpoints related to exposure of organisms to pollutants and xenobiotics are morphological changes and behavioral disturbances of varying severity and, ultimately, mortality [58,59,60]. Escape attempts, moving away from contaminated soil, remaining on the surface or attaching to test jar caps are some of the behavioral changes observed in earthworms [59,61]. Similarly, earthworms may show lethargy, increasing their hiding time in the soil or showing reduced reaction and movement to light and physical contact [62,63]. On the other hand, some morphological changes in earthworms are based on the expulsion of coelomic fluid (related to stress factors), mucus and bleeding [64,65]. In addition, coiling and uncontrolled muscle contractions can appear related to neurotoxicity [65,66]. Some xenobiotics cause severe injuries such as mutilations and loss of segments in a detoxification attempt [39,65,67]. Other injuries include deformation and clitellar swelling, tissue abrasion or pigmentation changes, among others [39,59,65].

In this study, after 14 days of exposure to the different concentrations of polyester resins R930A-SP and R930A-DVE1, the survival rate of earthworms was 100%. In daily observations and on days 7 and 14, none of the described morphological or behavioral changes was observed.

**Figure 1:**
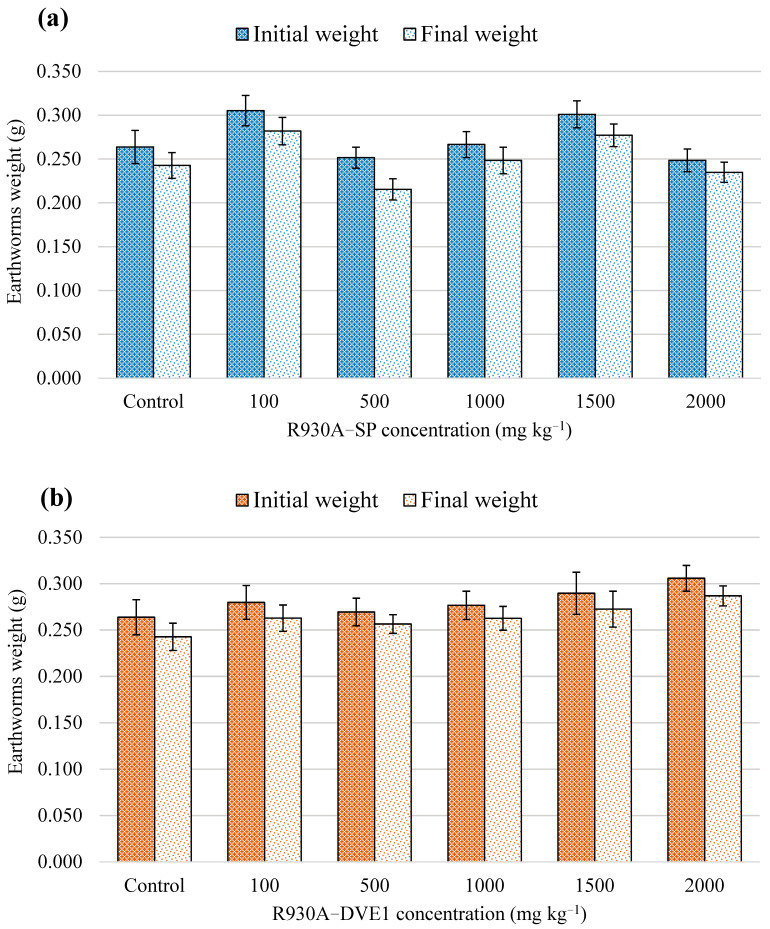
Earthworm weights measured for each treatment and control in R930A−SP (**a**) and R930A−DVE1 (**b**) polyester resins. The bars represent the mean ± SEM of the weights at the beginning and end of the test, after 14 days of exposure.

Another important morphological endpoint, especially for earthworms, is weight changes. In these organisms, weight loss is a direct indicator associated with detoxification mechanisms, energy metabolism, and response to stress due to exposure to pollutants and toxicants [59,60,68]. In this work, the weights at the beginning and end of the test, expressed as mean ± SD, were 0.278 ± 0.064 g and 0.258 ± 0.056 g, respectively. Earthworms’ growth inhibition (GI) [69] was 7.07 ± 0.09% (SD), calculated with the following formula: GI = (W0 − Wt)/W0 · 100, where W0 is the initial mean weight and Wt is the final mean weight after 14 days of exposure. The initial and final weights for each of the concentrations and the control for both resins are shown in Figure 1.

Statistical analyses indicated data were normal and homoscedastic. Dunnett’s test results showed no significant difference between the control and all treatments with both thermoset polyester resins. Therefore, calculated growth inhibition is not relevant.

The results obtained for weight changes and survival rate of earthworms exposed to both polyester resins are in line with results reported by other authors who have worked with environmentally relevant concentrations of different types of microplastics in earthworms. In this sense, Sobhani et al. [45] considered a range of environmentally relevant concentrations of polyethylene microplastics from 0.01 to 0.5% *w*/*w* in a chronic assay (up to 180 days of exposure) and no earthworm mortalities were reported at any of the concentrations studied. Similarly, Lackmann et al. [39] obtained no mortalities in earthworms (2, 7, 14 and 28 days of exposure) using polystyrene microplastics with a flame retardant (PS-HBCD) and car tire abrasion at environmentally relevant concentrations ranging from 0.00001 to 0.1% *w*/*w*. Likewise, according to Chen et al. [36], no mortalities or significant weight changes were recorded after 14 days of exposure of earthworms to an environmentally relevant polypropylene and polyethylene concentration of 0.2% *w*/*w*. Finally, Zhou et al. [70] conducted a high-concentration assay (from 0.03 to 0.9% *w*/*w*) of polypropylene microplastics on earthworms resulting in mortalities only after 42 days of exposure to PP concentrations > 0.3% *w*/*w* and significant growth inhibition for concentrations > 0.6% *w*/*w* after 14 days of exposure. Therefore, both thermoset polyester resins tested under the proposed experimental conditions did not appear to cause visible damage or alterations in the earthworms exposed. Additionally, resins tested seemed to behave in a similar way to environmentally relevant microplastic concentrations in terms of survival rate and weight changes.

### 3.2. Enzymatic Biomarkers

Subcellular level changes as early warning signals were assessed by three enzymatic biomarkers: cholinesterases (ChE), carboxylesterases (CbE) and glutathione S-transferases (GST).

**Figure 2:**
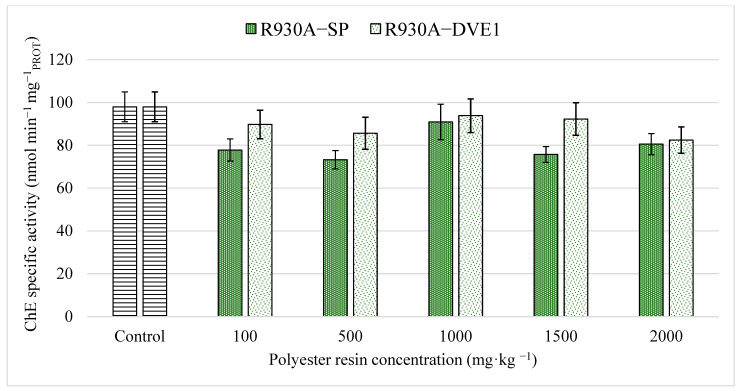
Cholinesterase specific activity in earthworms obtained for both polyester resins studied. The bars represent the mean ± SEM for each of the treatments and the control after 14 days of exposure.

ChE is commonly used as a biomarker to evaluate the neurotoxicity of a substance in different organisms [39]. Specifically for earthworms, ChE is considered a mechanism-specific biomarker for neurotoxicity [61], as it is associated with neuro-signaling, playing a vital role in the information transmission function in the nervous system of these organisms [43,61]. Figure 2 shows the specific activity obtained for ChE in this work after 14 days of exposure to the two thermoset compounds studied. Statistical analyses revealed non-significant differences between any of the treatments and with respect to the control (*p*-values: 0.456). Therefore, the polyester resins R930A-SP and R930A-DVE1 do not seem to cause neurotoxic effects in earthworms for the concentrations and exposure time considered.

The results obtained for thermoset compounds in terms of neurotoxicity are very consistent with results previously reported by other authors using earthworms with other plastic materials. Liang et al. [44] reported ChE inhibition in earthworms only at long exposures (30 days) using a mixture of polyethylene and cadmium. Zhong et al. [43] achieved ChE alterations in vermicomposting containing polyethylene at low concentrations, although at 20 to 80 days of exposure. Lackmann et al. [39] obtained highly significant effects on ChE only for exposures longer than 28 days with concentrations of 0.01% *w*/*w* polystyrene with flame retardant (PS-HBCD).

Moreover, CbE is considered an important biotransformation enzyme involved in the metabolism and detoxification of xenobiotics [39,59,60]. Concerning earthworms, CbE is considered remarkably involved in the metabolism and detoxification mechanisms of these organisms [53,71]. Indeed, as reported by He et al. [59], CbE activity in earthworms shows the slowest recovery rate compared to other organisms, making CbE a very suitable biomarker to assess the impact of pollutants on these soil organisms.

Analogous to CbE, GST is a detoxification enzyme involved in the metabolism of xenobiotics, in this case via conjugation with the glutathione system [39,60,61,71]. For many organisms as well as earthworms, GST is the most relevant phase II enzyme in the detoxification metabolism of a wide range of organic and inorganic compounds, suggesting GST is a suitable biomarker for chemical substances of different nature [65,72]. GST can remove reactive oxygen species (ROS) and malondialdehyde caused by oxidative stress and lipid peroxidation, accelerating the metabolism of pollutants [59]. Therefore, GST is also considered a biomarker of oxidative stress due to the metabolic reactions involved [50]. An interesting feature of GST, particularly in earthworms, is the increase in enzymatic activity to reduce ROS and xenobiotics [59,65]. However, GST can also inhibit or decrease enzymatic activity with an excess of ROS and free radicals, which affect the normal capacity of the earthworm’s repair and detoxification mechanisms [59].

**Figure 3:**
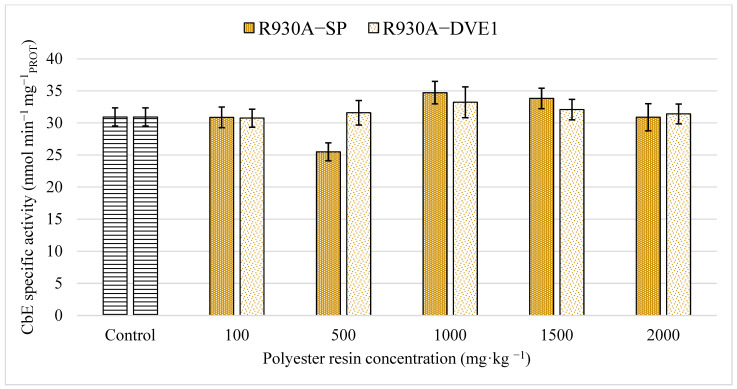
Specific activity of carboxylesterase in earthworms obtained for both polyester resins studied. The bars represent the mean ± SEM for each of the treatments and the control after 14 days of exposure.

**Figure 4:**
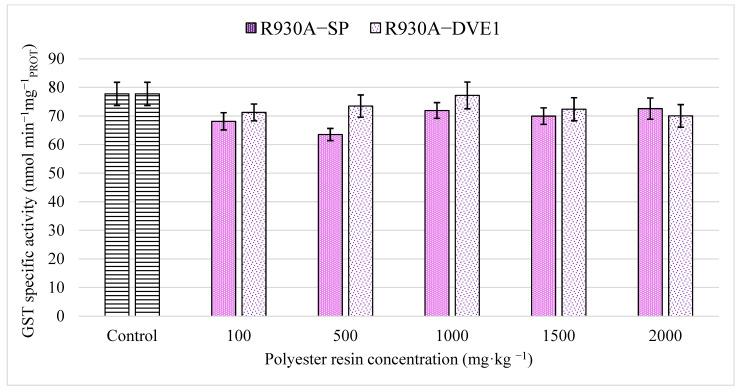
Specific activity of glutathione S-transferase in earthworms obtained for both polyester resins studied. The bars represent the mean ± SEM for each of the treatments and the control after 14 days of exposure.

Results of specific activity for CbE and GST biomarkers obtained in this study are shown in Figure 3 and Figure 4, respectively. In both cases, no significant differences were obtained between the treatment groups or between the treatment groups and the control group, according to the ANOVA test (*p*-values: 0.261 and 0.057, respectively). Based on the results obtained, both thermoset polyester resins do not appear to significantly affect the xenobiotics metabolism or cause significant oxidative stress in earthworms under the conditions tested.

Comparing the results obtained with other studies, Lackmann et al. [39] reported very similar results for CbE in earthworms exposed to microplastics, where no inhibition was observed. In this sense, no further studies concerning the effect of plastic compounds on CbE activity in earthworms were found. Similarly, the results obtained in this work for GST are also in agreement with other studies regarding oxidative stress in earthworms caused by plastic materials. Lackmann et al. [39] obtained no significant differences for GST in earthworms exposed to different microplastics with exposure times up to 28 days. Jiang et al. [73] reported very low oxidative stress in experiments with earthworms exposed to polystyrene concentrations of 0.0001% *w*/*w*. Liang et al. [44] found an increase in GST activity due to oxidative stress in earthworms at exposure times of 10 days but co-exposed to polyethylene and cadmium. Zhou et al. [70] stated that microplastics such as polypropylene might induce oxidative stress in earthworms at concentrations greater than 0.3% *w*/*w*. Cui et al. [74] reported inhibitions of GST activity and other biomarkers of oxidative stress in earthworms within 14 days but for polyethylene and polystyrene concentrations of 20% *w*/*w*.

Therefore, the adverse effect of plastic materials on earthworms seems to be strongly dependent on exposure time and concentration. As suggested by Cui et al. [74], microplastics in earthworms have no apparent effect on most biomarkers at low exposure levels and do not seem to induce oxidative stress and xenobiotic metabolism under most environmental conditions. In this regard, when simulating a possible future scenario with potentially relevant environmental concentrations, thermoset polyester resins under the conditions studied would seem to exhibit similar effects and behavior as other microplastics already present in the environment. Furthermore, according to Mondal et al. [75], such a wide range of plastic substances with different characteristics and properties makes the effects of microplastics on earthworms inconsistent and further in-depth studies are required. In fact, the toxicity of microplastics on earthworms seems to be more relevant when combined with metals and other organic compounds [74,76,77].

Finally, in this work, no significant differences were observed between thermoset resin with styrene (R930A-SP) and the more biodegradable thermoset resin formulation (R930A-DVE1). More research is needed on this topic, although these findings also are in line with other studies. Zhao et al. [78] and Baihetiyaer et al. [79] suggested that more biodegradable bio-based plastics were not significantly advantageous to conventional plastics in terms of their effects on enzymatic biomarkers and other endpoints in earthworms.

## 4. Conclusions

In this work, an evaluation has been carried out, for the first time, of the effects on enzymatic biomarkers and other endpoints in earthworms of two thermosetting polyester resins with different formulations, one conventional and the other more biodegradable. Experimental conditions proposed were based on a realistic premise simulating potentially environmentally relevant concentrations and were compared with studies on other plastics already present in the environment. At concentrations and exposure times studied, no significant effects on growth, measured as weight loss, or on the enzyme biomarkers ChE, CbE and GST were observed. Similarly, no behavioral changes were detected in the earthworms, and the survival rate was 100%. Likewise, no differences were observed between the different formulations of the polyester resins studied.

Nevertheless, wide knowledge gaps still exist, and more in-depth studies are required. Chronic studies and analyses of more biomarkers and other endpoints such as reproductive effects are suggested, determining a potential dependence of the adverse effects of resins on exposure time, as shown in other studies with microplastics. Moreover, it is important to understand how these compounds will behave under changing climatic conditions and to conduct toxicity tests on these aged materials. In addition, studies should be carried out on other representative organisms from different environmental compartments considering the potentially ubiquitous nature of thermosets in the short term. Similarly, further research on the combined effects of thermosets with other pollutants such as metals and organic compounds already present in the environment is also suggested. Alternatively, effects of higher concentrations of thermoset polyester resins might be investigated in order to understand and assess a potential risk of these materials, despite compromising relevance or realism from an environmental point of view.

It is true that any environmental study can be complex, and this must be kept in mind. Potential limitations of these studies may lie in the difficulty of determining the degradation products of resins once released into the environment and the interactions they may have with other compounds present in the environment. These conditions are difficult to reproduce in a laboratory.

## Data Availability

The data presented in this study are available on request from the corresponding author due to specifications of the BIZENTE project.

## Abbreviations

The following abbreviations are used in this manuscript:

MEKP	Methyl ethyl ketone peroxide
VOC	Volatile organic compound
HAP	Hazardous air pollutant
IARC	International Agency for Research on Cancer
ChE	Cholinesterase
CbE	Carboxylesterase
GST	Glutathione S-transferase
GSH	L-Glutathione
DTNB	5,5′-Dithiobis (2-nitrobenzoic acid)
CDNB	1-Chlro-2,4-dinitrobenzene
PS-HBCD	Polystyrene with flame retardant
PP	Polypropylene
ROS	Reactive oxygen species
